# Intraocular pressure in a cohort of healthy eastern European schoolchildren: variations in method and corneal thickness

**DOI:** 10.1186/1471-2415-12-61

**Published:** 2012-12-02

**Authors:** Patrycja Krzyżanowska-Berkowska, Magdalena Asejczyk-Widlicka, Barbara Pierscionek

**Affiliations:** 1 Department of Ophthalmology, Wroclaw Medical University, Borowska 213, 50-556, Wroclaw, Poland; 2 Institute of Physics, Wroclaw University of Technology, Wybrzeze Wyspianskiego 27, 50-370, Wroclaw, Poland; 3 Faculty of Science, Engineering and Computing, Kingston University, (Roehampton Vale Campus), Friars Avenue, London, SW15 3DW, UK

**Keywords:** Cornea, Child, Paediatric Ophthalmology, Intraocular pressure, Tonometry

## Abstract

**Background:**

Intraocular pressure (IOP) in the developing eye of a child is not always easy to measure and there is no technique that is known to be the most accurate for the young eye. Measurements are needed on many cohorts of children with different tonometers to determine how the values correlate between instruments, whether corneal parameters affect readings and whether correlations between age and IOP values can be discerned. The aim of this study was to undertake a comparative analysis of three different tonometers on a group of healthy children to see whether differences exist and whether these may be related to central corneal thickness and/or radius of curvature. In addition, the study adds to the relatively small body of literature on IOP in the growing eye which will collectively allow trends to be identified and ultimately norms to be established.

**Methods:**

IOP was measured on 115 eyes in a group of Polish children, aged between 5–17 years (mean ± standard deviation [SD] 11.3 ± 3.0 years) using three different tonometers: non-contact (NCT), the ICare and Goldmann applanation (GAT). Readings obtained were compared between instruments and with central corneal thickness and radius of curvature.

**Results:**

The ICare tonometer provided statistically higher IOP values (16.9 ± 3.4 mmHg) than the GAT (14.7 ± 2.9 mmHg) regardless of corneal thickness and whether or not a correction factor was applied. A correlation was found between central corneal thickness (CCT) and IOP values obtained with all three tonometers but only the IOP values detected with the ICare tonometer showed a statistically significant correlation with radius of curvature (p < 0.004). No correlations with age or gender were found for IOP values measured with any of the instruments.

**Conclusions:**

IOP measurements on children vary significantly between instruments and correlations are affected by the corneal thickness. Further studies on children are needed to determine which instrument is most appropriate and to derive a normative IOP scale for the growing eye.

## Background

An accurate, reliable and repeatable measure of intraocular pressure (IOP) is one of the fundamental requirements for glaucoma diagnosis and applanation tonometry remains the most commonly used and trusted technique in clinical practice. In children, however, standard methods such as applanation tonometry may be difficult to administer and consequently yield inaccurate or erroneous results [1-6].

Whilst a number of studies comparing IOP measurements with the ICare tonometer with applanation tonometry and other methods have been published, these have been conducted mainly in adults, a proportion of whom were patients diagnosed with glaucoma [7-13]. A relatively small proportion of the literature is devoted to measurements of IOP in children. Even fewer studies have described measurements, on young eyes, using the ICare tonometer.

The growing and developing eye in a child is different compared to the fully grown and therefore more stable eye of the adult. Consequently, the standard normal range of IOP in adults cannot be used for children. Measurements on large populations of children, without eye disease or conditions that may affect IOP, from a variety of countries and ethnic groups is required, to determine the standard range of IOP values applicable for every age of the growing eye.

The measurement of IOP in children yields difficulties that are not associated with tonometry on adult eyes. In addition to the issues that relate to attention, understanding of the procedure and co-operation with the examination, that can render contact methods difficult and measurements inaccurate, there are the physiological differences in ocular biometry and its proportions between the adult system and growing eyes of children.

The aim of this study was to evaluate the IOP values in a large group of children without systemic or ocular diseases and to investigate the effect of central corneal thickness (CCT) and radius of curvature on the IOP values measured. The cohort was an ethnically homogenous Polish population and three methods were used to collect IOP readings. The values for each method were compared with each other and with biometric data: CCT and radius of curvature.

## Methods

The subject base consisted of 75 children (30 female and 45 male) with a low refractive error range (± 2D (sphere); ± 0.5D (cylinder)), aged between 5 and 17 years (mean ± standard deviation [SD] 11.3 ± 3.0 years) who presented for a regular eye examination. Measurements were made on 115 healthy eyes. Some of the subjects presented with ocular trauma in one eye and in such cases only the other, healthy, eye was included in the study. Only subjects who could comply with instructions given and from whom reliable measurements could be taken were used in this study. Subjects and their parents or guardians were fully informed of all procedures and why these were being conducted. In addition, subjects were carefully instructed, prior to each measurement, in what would be required of them during that procedure, the correct gaze position to adopt and, in the case of tonometry asked not to blink during the measurement. For measurements where contact with the eye was necessary, this was first demonstrated on the hand of the subject to indicate the level of contact that would be made on the eye.

Visual acuity was measured at distance and near with and without correction (BCVA). Radius of curvature was measured using an autorefraktometr Speedy-K (Righton). Three tonometers were used to obtain IOP: a non-contact tonometer (NCT) (Reichert, ophthalmic instrument XPERT NCT Plus Advanced Logic Tonometer), the ICare tonometer (TA01i, Icare Finland Oy) and the Goldmann applanation tonometer (GAT) fitted to a Nikon Slit Lamp NS-1 V. After measuring the radius of curvature, IOP was measured with the NCT and then with the ICare tonometer without anaesthetic drops. One drop of 0.5% Alcaine (Proparacaine Hydrochloride, Alcon-Couvreur) was instilled into the examined eye prior to measuring CCT. One drop of 2% Thilorbin (Fluorescein Sodium, Oxybuprocaine Hydrochloride, Alcon Pharma GmbH) was then put into the conjunctival sac, 30 seconds before the GAT measurement. Three NCT measurements, three ICare measurements and three GAT measurements were taken for each eye in the study. For each tonometer, values were averaged and the averages used for analysis. Variations in readings, for a given subject were NCT (± 2 mmHg), ICare (± 1 mmHg) and GAT (± 1 mmHg). Measurements were rendered invalid if a subject blinked or moved their eye or head. At least quarter of an hour was taken between successive measurements with the different instruments in order to exclude any residual effect of previous measurements.

Six measurements of CCT were taken for each eye with an ultrasound pachymeter (Pacline Optikon 2000 OKB 181). The average values, within ± 5 μm, were accepted for analysis. All measurements were made between 9 am and 11 am.

Statistical analysis was conducted using STATISTICA 7 (StatSoft, Inc.). The three methods of IOP measurements were compared using the analysis of variance (ANOVA) and Tukey’s HSD test. Equality of variances was tested using Brown–Forsythe and Levene’s tests and the Pearson product–moment correlation coefficient (*r*) was used to determine correlations between variables (*r* = 0.7-0.99- high correlation, *r* = 0.4-0.69- medium correlation and *r* <0.4 - no correlation). Statistical significance was taken as p < 0.05.

The project was approved by the Ethics Committee of the Faculty of Ophthalmology at the Medical Academy in Wrocław and adhered to the Tenets of the Declaration of Helsinki. Informed parental consent and the assent of the subject were obtained before measurements were taken.

## Results

Subject age, CCT, radius of curvature and IOP results, as measured with the three tonometers, are shown in Table 1 for the entire data set and for subsets with thinner and thicker corneae. No trend or correlation was found between age and IOP with any of the instruments.

**Table 1:** Biometric and clinical data for the entire cohort, and for subgroups with central corneal thickness (CCT) < and ≥563 μm

	**All**	**CCT < 563 μm**	**CCT ≥ 563 μm**
	**mean ± SD**	**mean ± SD**	**mean ± SD**
age [years]	11.3 ± 3.0	11.9 ± 2.8	10.7 ± 3.0
CCT [μm]	563 ± 30	540 ± 18	588 ± 19
R [mm]	7.85 ± 0.29	7.82 ± 0.30	7.87 ± 0.28
ICare [mmHg]	16.9 ± 3.4	15.8 ± 2.6	18.1 ± 3.7
GAT [mmHg]	14.7 ± 2.9	13.6 ± 2.6	15.8 ± 2.8
NCT [mmHg]	15.9 ± 3.5	14.4 ± 3.0	17.6 ± 3.4

**Figure 1:**
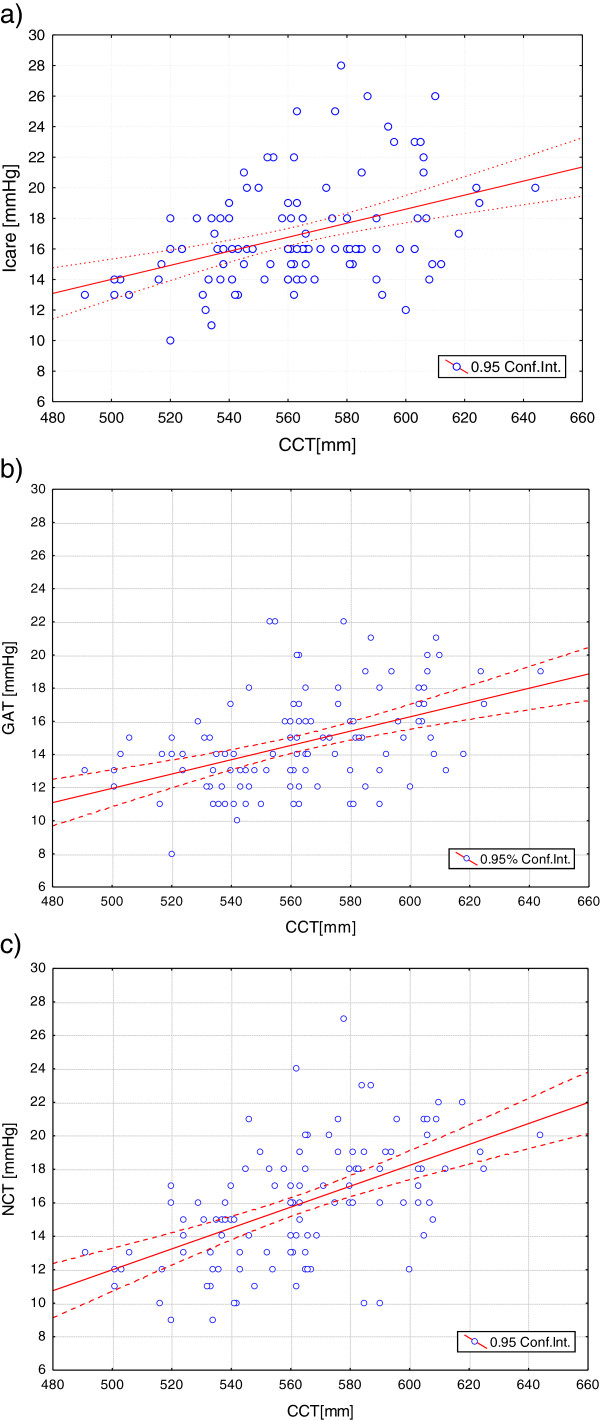
Intraocular pressure (IOP) plotted against central corneal thickness (CCT) (μm) for IOP values obtained with the a) ICare tonometer; b) Goldmann applanation tonometer (GAT); c) non-contact tonometer (NCT).

The lowest IOP values were obtained with the GAT (14.7 ± 2.9 mmHg); the highest with the ICare tonometer (16.9 ± 3.4 mmHg). When the IOP results are plotted against the CCT (Figure 1), there is a positive linear trend that is steepest for the NCT. The variation of IOP with CCT was highly statistically significant for all three tonometers (ICare, p < 0.001; GAT, p < 0.001; NCT, p < 0.001). Whilst CCT is highly correlated with IOP, only IOP values obtained with ICare tonometer have a statistically significant correlation with radius of curvature (p < 0.004).

**Figure 2:**
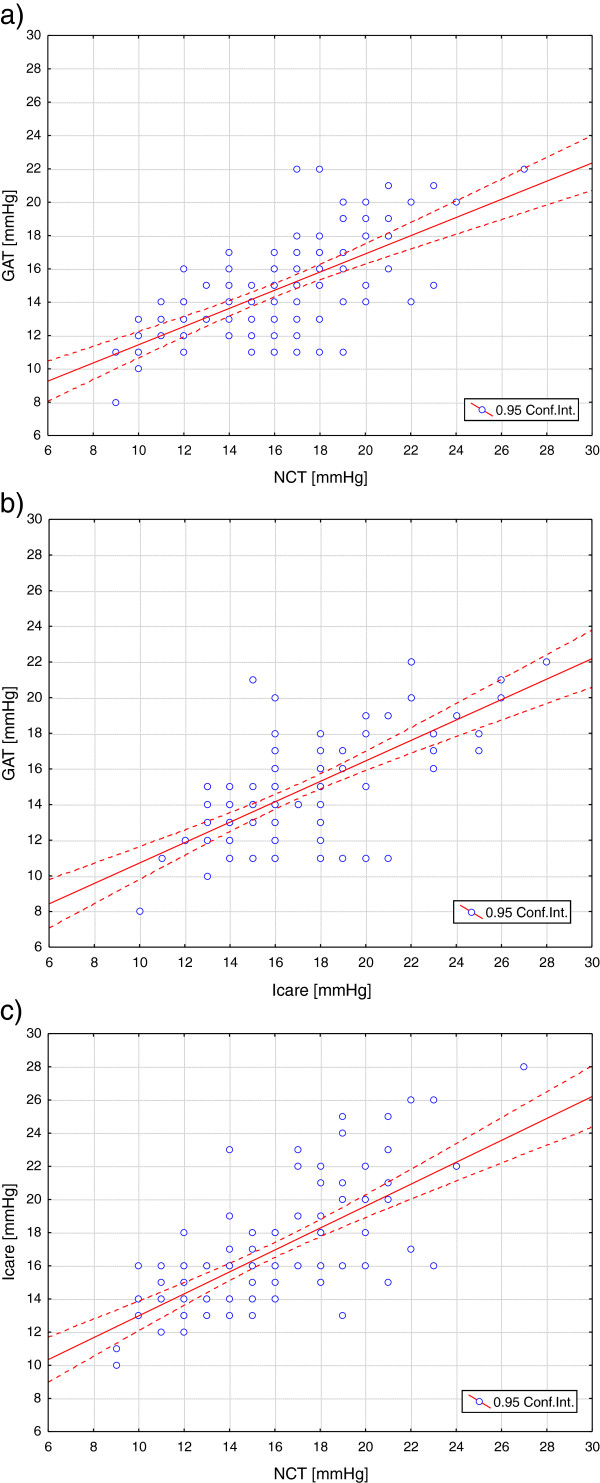
Intraocular pressure (IOP) obtained with a) Goldmann applanation tonometer (GAT) plotted against those obtained with the non-contact tonometer (NCT); b) Goldmann applanation tonometer (GAT) plotted against those obtained with the ICare tonometer; c) the ICare tonometer plotted against those obtained with the non-contact tonometer (NCT).

To determine whether measurements of IOP obtained from the three instruments, differed significantly, comparative plots of the NCT and GAT, the ICare and the GAT and the NCT and ICare were plotted as shown in Figures 2a), b) and c) respectively. When confidence intervals (95%) are compared for the whole cohort: NCT (CI 15.26-16.60), ICare (CI 16.26-17.54), GAT (CI 14.13-15.23), a statistically significance difference, based on ANOVA and Tukey HSD testing was found between GAT and ICare (p < 0.001) and GAT and NCT (p = 0.01), but not between NCT and the ICare tonometer (Table 2).

**Table 2:** Correlations between tonometers: ICare, Goldmann applanation tonometer (GAT) and non-contact tonometer (NCT) for the entire cohort, and for subgroups with central corneal thickness (CCT) < and ≥563 μm

	**All**	**CCT < 563 μm**	**CCT ≥ 563 μm**
	**p**	**p**	**p**
Icare vs. GAT	**<0.001**	**<0.001**	**<0.001**
Icare vs. NCT	0.09	**0.02**	0.85
GAT vs. NCT	**0.01**	0.48	**0.01**

In order to investigate the effect of CCT on the comparability of IOP measurements between different tonometers, the cohort was divided into two groups depending on CCT range: either side of the mean value of 563 μm: < 563 μm (59 subjects) and ≥ 563 μm (56 subjects) (Table 1). A slightly greater range of IOP values with the three tonometers is seen for the group with the thicker corneae. Comparison of confidence intervals (CI: GAT: 12.97-14.33; NCT: 13.58-15.14; ICare: 15.12-16.48) shows that for thinner corneae (<563 μm), there is a statistically significant difference between IOP values taken with the GAT and ICare tonometer (p < 0.001) and between those obtained with the NCT and ICare (p = 0.02) but not between measurements obtained with GAT and NCT (p = 0.48) (Table 2). For the group with the higher CCT, statistical significance is found between ICare and GAT (p < 0.001) and GAT and NCT (p = 0.01) but not between ICare and NCT (p = 0.85) (Table 2) (CI: GAT: 14.12-15.22; NCT: 15.27-16.58; ICare: 16.38-17.54). There is no statistically significant correlation between radius of curvature and IOP values obtained with any instrument for the group with thinner corneae but a correlation between radius of curvature and IOP obtained with the ICare tonometer is found for the group with the thicker corneal values (p < 0.008). For the cohort with thinner corneae, CCT was statistically significantly correlated with IOP obtained with the ICare tonometer and the NCT (p < 0.006, p <0.002 respectively). Conversely, for the group with thicker corneae, correlation with CCT was found only with the GAT (p < 0.025).

The IOP measures obtained with the GAT can be corrected for thickness differences, using the Ehlers correction factor, an algorithm derived for the adult eye [14]. Applying the correction factor, does not alter the correlations between IOP values obtained with the different tonometers. For the entire cohort as well as for the subgroup with CCT ≥ 563 μm, statistically significant differences were found between IOP measurements obtained with the GAT and measurements obtained with both of the other instruments; for the subgroup with CCT < 563 μm, measurements taken with ICare tonometer are statistically significantly different to those with either of the other instruments.

**Figure 3:**
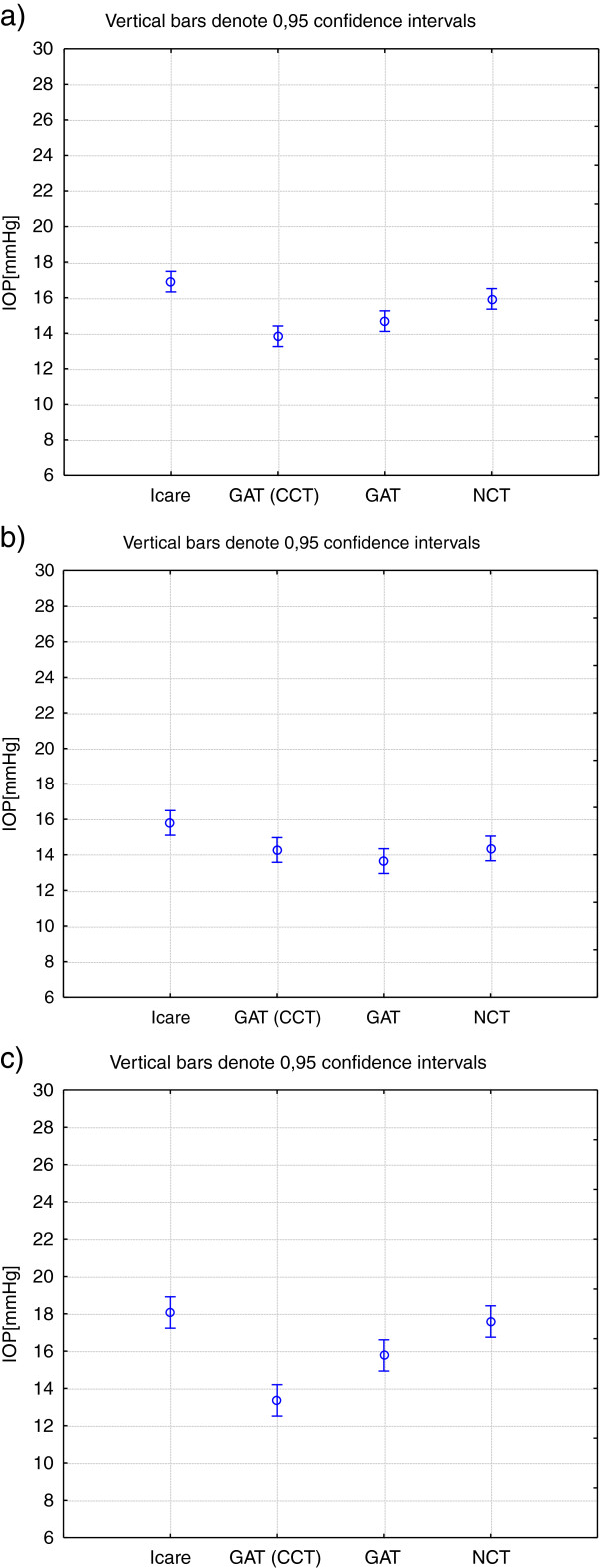
Confidence intervals for intraocular pressure values (IOP) (mmHg) obtained with the ICare tonometer, Goldmann applanation tonometer (GAT) corrected and uncorrected and the non-contact tonometer (NCT) for a) the entire cohort; b) subgroup with CCT <563 μm; c) subgroup with CCT ≥ 563 μm.

Figure 3 shows the confidence intervals for IOP values measured with the ICare tonometer, GAT (corrected and uncorrected) and for the NCT, for the entire cohort (Figure 3a), for the subgroup with CCT < 563 μm (Figure 3b) and for the subgroup with CCT ≥ 563 μm (Figure 3c). It is notable that for the latter, there is a significant difference between the corrected and the uncorrected GAT values.

When the data are divided into lower and higher ranges of radius of curvature, taking the mean of 7.85 mm as the midpoint (Table 3), statistically significant differences are found only between IOP measurements obtained with the ICare instrument and the GAT for cohorts of both lower (<7.85 mm) and higher (≥7.85 mm) radii of curvature (Table 4).

**Table 3:** Biometric and clinical data for the entire cohort, and for subgroups with corneal radius (R) < and ≥ 7.85 mm

	**All**	**R < 7.85 mm**	**R ≥ 7.85 mm**
	**mean ± SD**	**mean ± SD**	**mean ± SD**
age [years]	11.3 ± 3.0	11.4 ± 3.0	11.2 ± 3.0
CCT [μm]	563 ± 30	559 ± 18	568 ± 17
R [mm]	7.85 ± 0.29	7.63 ± 0.15	7.09 ± 0.20
ICare [mmHg]	16.9 ± 3.4	15.9 ± 2.8	18.0 ± 3.6
GAT [mmHg]	14.7 ± 2.9	14.3 ± 2.4	15.1 ± 3.4
NCT [mmHg]	15.9 ± 3.5	15.5 ± 3.3	16.5 ± 3.7

**Table 4:** Correlations between tonometers: ICare, Goldmann applanation tonometer (GAT) and non-contact tonometer (NCT) for the entire cohort, and for subgroups with corneal radius (R) < and ≥7.85 mm

	**All**	**R < 7.85 mm**	**R ≥ 7.85 mm**
	**p**	**p**	**p**
Icare vs. GAT	**<0.001**	**0.009**	**<0.001**
Icare vs. NCT	0.09	0.85	0.07
GAT vs. NCT	**0.01**	0.09	0.17

**Figure 4:**
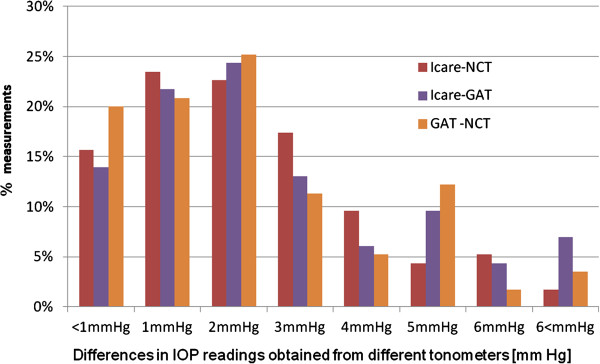
Percentage of measurements plotted against varying differences in IOP readings between the three instruments tested.

The differences in IOP values obtained from the three tonometers were not normally distributed and hence Bland-Altman plots were not constructed. The proportion of measurements (as a percentage of the whole set) showing differences in magnitudes of IOP are shown plotted in Figure 4. For all three data pairs, in over 60% of measurements, differences in IOP values were  ≤ 2 mmHg. Around 20% of the measurements show differences in IOP, between ICare and NCT, of ≥ 4 mmHg. This compares to 23% of measurements showing differences of such magnitude between GAT and NCT and 26% showing this magnitude of difference between the ICare tonometer and GAT. There was no trend or consistency in the magnitude of the differences for any pair of tonometers.

## Discussion

A number of comparative analyses of tonometers have been conducted on adult eyes, both normal and glaucomatous or hypertensive [9-12]. Fewer studies have conducted similar comparative analyses with the eyes of children. The ICare tonometer was developed for use on patients who present with the sort of measurement problems that are often associated with children. The instrument is easy to handle, portable, light weight, and does not require anaesthesia or fluorescein. Some of these advantages are also pertinent to the NCT. Comparison of the ICare with an NCT on children from 6 months to 15 years of age showed comparability between instruments but also demonstrated the greater tolerance for measurements taken with the ICare tonometer compared with the NCT, on younger children [6]. High levels of tolerance for, as well as reproducibility of, IOP measurements were also found with the ICare tonometer, on a group of healthy infants [5] and schoolchildren [3]. Ease of use and tolerance, however, need to be balanced against effectiveness and accuracy. For many years, applanation tonometry was considered to set a standard against which other instruments were measured. However, recent reviews that have compared a number of instruments suggest that depending on the population and the application, different instruments may be preferred [15,16].

A previous study of 460 children aged from 0 to 16 years, found that IOP increases from birth to around 7–8 years (slightly earlier for males than for females) and then stabilises [2]. The results of this study do not show any correlation or trends with age but the population used in this study was largely beyond the age range over which the rapid increase in IOP was observed [2].

The findings indicate that radius of curvature is correlated with IOP only for the ICare tonometer; this applies for the entire cohort and for the subgroup of subjects with thicker corneae. The only other comparable study, on Turkish schoolchildren, aged between 7 and 12 years, found no correlation between IOP and radius of curvature [4]. Significant differences between measurements obtained with the ICare tonometer and GAT that exist for the entire data set are evident for subsets of both lower and higher radii of curvature ranges. Given that only results obtained using the ICare tonometer showed a statistically significant correlation with radius of curvature and that the most significant differences between tonometers for the whole data set are evident between the ICare tonometer and GAT, it is not surprising that measurements vary significantly between these two instruments in cohorts with lower and higher radii of curvature ranges. Previous findings on adult eyes have found radius of curvature to influence readings with GAT [17] and with ICare (for CCT > 556 μm) [18].

Correlation with CCT and IOP was found for all tonometers used and CCT was found to affect correlations between the IOP values obtained with the different instruments. These correlations applied whether or not a correction factor for the GAT was used. This correction factor, derived for an adult eye [14], may not be appropriate for the eyes of children.

It should be noted that the extent of the effect of CCT on IOP measurements is not clear even for the adult eye. Originally, Ehlers and colleagues proposed a correction factor for CCT variations based on finding a significant correlation between CCT and IOP [14]. However, subsequent studies have not been consistent in their findings about how, and indeed whether at all, CCT should be corrected. Whilst a number of studies have found variations in IOP with CCT [19-23], correction factors have varied [14,23] and the use of any correction factor for the healthy adult cornea has been questioned [24,25]. Paucity in the understanding of the rheological properties of the cornea prevents definitive conclusions from being made about the effect of CCT on IOP measurements in the adult eye.

This notwithstanding, the findings in this study indicate that the thickness of the cornea in children can have an effect on correlation of IOP measurements between instruments. The ICare tonometer and the GAT gave readings that were significantly different statistically regardless of CCT; the NCT gave comparable readings with the ICare tonometer for the subgroup with thicker cornea and for the group as a whole and was comparable with the GAT for the subgroup of CCT < 563 μm.

This is the first study of IOP measurements on healthy Polish schoolchildren. It contributes to the literature on IOP in the young eye and provides a comparative study to those conducted on other ethnic groups. Findings from this study show that average CCT values were higher than those found in controls aged between 5-17 years from the USA (mean CCT = 555 ± 37 μm) [26]. This included Caucasian and African ethnicities. For the Caucasian (‘white’) group alone, the average CCT was 564 ± 28 μm, which is very close to the mean value found in this study (563 ± 30 μm). The IOP for controls (Caucasian and African) was found to be 14.9 ± 2.7 mmHg for the Goldmann and 15.1 ± 2.4 mmHg for the Tono-Pen [19]. This compares to 14.7 ± 2.9 mmHg (GAT) in this paper. A study of 460 Italian subjects, aged 0 to 16 years of age, (with 282 subjects aged 5–16 years), showed a mean IOP of 13.9 ± 2.3 mmHg and 14.88 ± 2.39 mmHg for 10–16 year old males and females respectively using an NCT. These values are slightly lower than the IOP values found with the NCT in this study (15.9 ± 3.5 mm Hg, males and females combined) but are within the same range. No statistically significant differences between males and females were found in this study.

Previous investigations on 165 Turkish schoolchildren, aged 7–12 years, showed mean IOP values of 16.81 ± 3.1 mmHg using rebound tonometry which is very close to the 16.9 ± 3.4 mmHg found for the ICare in this study. IOP measurements on Japanese subjects aged from 6 months to 15 years of age reported mean IOP values of 15.9 ± 2.3 mmHg and 15.1 ± 2.6 mmHg in right and left eyes of 130 subjects respectively for the NCT with 15.1 ± 2.6 mm Hg and 13.9 ± 2.9 mmHg in right and left eyes respectively of 160 subjects for the ICare tonometer [6]. No explanation was given for the greater difference between right and left eyes using the latter instrument but the authors indicate that ICare is the more suitable instrument for children under 6 years of age [6].

A study that uses young subjects, notwithstanding the steps taken to explain the procedure and to take carefully controlled measurements, can be limited by the extent of subject participation and parental involvement. Collaboration of subjects, such as children, is greatly improved if the subjects feel comfortable in the environment where measurements are taken and if there is no or minimal parental pressure and/or anxiety. The University clinic where the study was conducted is one which routinely deals with large numbers of young subjects. It is purposely equipped to create an appropriate ambience and a relaxed atmosphere for these age groups. In addition, the subjects who participated in this study were all familiar with the clinic, parents/guardians had confidence in the investigators rendering high both subject and parental levels of cooperation.

The differences in IOP measurements from the three tonometers, presented in pairs (Figure 4), varied, for most measurements taken, by up to 2 mmHg. However, from a fifth to a quarter of the measurements varied by ≥ 4 mmHg. As there is no gold standard for tonometry measurement in children it is not possible to say which measurement can be taken as the most reliable. Bland-Altman plots, which are commonly used to check agreement between methods, often depend on one technique being the standard against which the other is measured [27]. Given the effect of CCT on IOP measurements and the paucity of knowledge about the growing eye or how to correct or adjust for CCT in such cases, it is prudent to avoid reliance on any one method. Many more studies on children from different cohorts are required before consistent trends can be established and expected age-related norms derived.

## Conclusions

There is still no universally accepted means of determining IOP in the growing eye. Three tonometers, used to obtain IOP values on a group of Polish children aged 5–17 years (mean ± SD 11.3 ± 3.0 years), show statistically significant differences in measurements. The ICare tonometer provides statistically higher IOP values than the GAT whilst correlations with the NCT and the other two instruments are affected by corneal thickness. Further studies on cohorts of children from different ethnic groups and using a range of instruments are needed before it is possible to determine which instrument is best for a growing eye and before a normative scale of IOP values for different stages of growth can be derived.

## Competing interests

The authors declare that they have no competing interests.

## Authors’ contributions

All authors conceived of and designed the experimental protocol. MAW and PKB collected the data. All authors were involved in the analysis. BP wrote the first draft of the manuscript. MAW and BP reviewed and revised the manuscript and produced the final version. All authors read and approved the final manuscript.

## Pre-publication history

The pre-publication history for this paper can be accessed here:

http://www.biomedcentral.com/1471-2415/12/61/prepub
